# Identification of a novel *FERMT1* variant causing kindler syndrome and a review of the clinical and molecular genetic features in Chinese patients

**DOI:** 10.3389/fped.2024.1425030

**Published:** 2024-09-06

**Authors:** Qiang Zhang, Qi Yang, Fei Shen, Linlin Wang, Jingsi Luo

**Affiliations:** ^1^ Laboratory of Genetic Metabolism Center, Maternal and Child Health Hospital of Guangxi Zhuang Autonomous Region, Nanning, China; ^2^ Guangxi Key Laboratory of Precision Medicine for Genetic Diseases, The Maternal and Child Health Care Hospital of Guangxi Zhuang Autonomous Region, Nanning, China; ^3^ Guangxi Key Laboratory of Reproductive Health and Birth Defect Prevention, The Maternal and Child Health Care Hospital of Guangxi Zhuang Autonomous Region, Nanning, China; ^4^ Guangxi Clinical Research Center for Pediatric Diseases, The Maternal and Child Health Care Hospital of Guangxi Zhuang Autonomous Region, Nanning, China; ^5^ Hematology Laboratory, Sheng Jing Hospital of China Medical University, Shenyang, China

**Keywords:** kindler syndrome, *FERMT1* gene, novel variation, whole exome sequencing, genetic analysis

## Abstract

**Background:**

Kindler Syndrome (KS, OMIM #173650), a rare autosomal recessive genetic disorder, is characterized by a spectrum of symptoms such as cutaneous fragility, blistering, photosensitivity, and mucosal involvement. These symptoms result from variations in the *FERMT1* gene (Fermitin family member 1, OMIM: 607900), encoding kindlin-1, an essential component of focal adhesions.

**Objective:**

This study aims to ascertain the potential pathogenicity of a *FERMT1* variant identified in a Chinese patient and to explore the phenotypic and molecular genetic characteristics of all reported cases of Kindler Syndrome in the Chinese population.

**Methods:**

Whole-exome sequencing (WES) was performed on the patient to identify candidate variants associated with KS, and Sanger sequencing was utilized to authenticate their presence and origin. To further assess the potential impact of these genetic variants, we employed a variety of in silico prediction tools. Concurrently, a review of various databases was undertaken to ascertain and consolidate information regarding cases of KS in Chinese families.

**Results:**

We identified a novel likely pathogenic frameshift variant in the *FERMT1* gene, specifically c.567_579delTATATATGACCCC (p.Ile190Serfs*10). The clinical presentation of this patient aligns with the diagnostic criteria for KS. The literature review reveals that the core clinical features of KS reported in the Chinese population include skin abnormalities (100%), as well as hyperkeratosis of the palms and soles (91.70%). Other clinical phenotypes encompass nail abnormalities (77.78%), abnormalities of the fingers/toes (75.00%), oral damage (70.00%), eye abnormalities (57.14%), and constipation (50.00%).

**Conclusion:**

Our study enriches the genetic landscape of KS in the Chinese population and augments the understanding of phenotypic variability resulting from *FERMT1* gene variants. The findings hold considerable significance for refining variant-based screening, genetic diagnosis, and comprehending the molecular pathogenesis underlying *FERMT1*-related disorders.

## Introduction

Kindler Syndrome (KS), initially described in 1954, is a rare autosomal recessive genetic disorder. The exact prevalence is not well-established. However, estimates for the United States, Europe, Asia, and Australia suggest a range from approximately 19 cases per 1 million live births to 11 cases per 1 million individuals in the general population (1). To date, over 400 cases of KS, resulting from variants in the *FERMT1* gene, have been documented (2). KS was recognized as a distinct entity within the Epidermolysis Bullosa (EB) spectrum during the 2003 Third International Consensus on Diagnosis and Classification of EB, based on its unique clinical and biological characteristics (3, 4). It is considered one of the rarest subtypes within the EB family, characterized by its distinctive clinicopathological and molecular abnormalities (5). KS is caused by variations in the *FERMT1* gene (formerly KIND1 or C20orf42), which is located on chromosome 20p12.3 (6). *FERMT1* encodes kindlin-1 (fermitin family homolog-1, FFH1), a protein expressed ubiquitously in various tissues, including the skin, periodontal tissues, and gastrointestinal tract (7). Kindlin-1 is an integral component of the basal keratinocyte cytoskeleton and plays a pivotal role in integrin signaling. It mediates integrin activation, links the actin cytoskeleton to the extracellular matrix, and is crucial for cell adhesion (8–11). Kindlin-1 plays a crucial role in maintaining keratinocyte proliferation, polarization, and structural integrity; it is required for proper adhesion of keratinocytes to extracellular matrix components such as fibronectin and laminin, as well as for their migration during wound healing (12, 13).

Clinically, KS is typically marked by bullae induced by trauma or sunlight, diffuse cutaneous atrophy, and early-onset poikiloderma (14). While photosensitivity often diminishes with age, there is an increased propensity for the development of malignant skin tumors in adulthood (15, 16). Mucosal involvement usually becomes apparent during adolescence, with the oral cavity being particularly vulnerable, demonstrated by symptoms such as hemorrhagic gingivitis, periodontitis, and premature tooth loss. Conjunctivitis, ectropion, esophageal stenosis, urethral strictures, anal stenosis, and severe colitis are other manifestations (4, 9).

In this study, we describe the case of a Chinese boy presenting with extensive poikiloderma, progressive xerosis, cutaneous atrophy, abnormal photosensitivity, and gingival hypertrophy. These symptoms were traced to a novel variant in the *FERMT1* gene.

## Materials and methods

### Next-generation sequencing

Genomic DNA was extracted from the patient and used to prepare a sequencing library. The library was prepared using the Agilent SureSelect Clinical Research Exome V2 Kit (Agilent Technologies, Santa Clara, CA, USA). Sequencing was performed on the Illumina HiSeq2500 System (Illumina, San Diego, CA, USA). Sequencing reads were aligned to the human reference genome (GRCh37/hg19) using the Burrows-Wheeler Aligner (BWA) software (v0.7.15). Variant calling and annotation were performed using the Genome Analysis Toolkit (GATK), followed by further annotation refinement and variant prioritization with TGex software (v5.7, LifeMap Sciences).

### Sanger sequencing confirmation

To validate candidate variants identified using TGex software and ascertain their inheritance patterns, Sanger sequencing was performed. For this purpose, primers were designed with Oligo7 software and subsequently synthesized by Sangon Biotech, located in Shanghai, China. Specifically, the primers 5'-ACAATTCTCCAACCTCAGCC-3’ and 5'-CGTGACATCCCATCTCTTAC-3’ were used to amplify the region of the *FERMT1* gene harboring the c.567_579delTATATATGACCCC (p.Ile190Serfs*10) variant.

### Bioinformatic analysis and verification of observations

We employed a suite of predictive tools to assess the functional impact of the identified variations (Figure 2B). This suite includes Mutation Taster (https://www.mutationtaster.org/), Provean (http://provean.jcvi.org/seq_submit.php), ENTPRISE-X (http://cssb2.biology.gatech.edu/entprise-x), and the Variant Effect Scoring Tool (https://www.cravat.us/CRAVAT/). These tools together facilitate a comprehensive analysis from multiple perspectives. For the categorical classification of the variant, we followed the guidelines established by the American College of Medical Genetics and Genomics (ACMG) and the Association for Molecular Pathology (AMP), as elaborated in the authoritative publication by Li and Wang (17).

## Results

### Clinical presentation

A 12-year-old boy from China visited our dermatology clinic, presenting with significant skin peeling (desquamation) and swollen gums. Born at full term to healthy, non-consanguineous parents, his birth and pregnancy were uneventful. Shortly after birth, blisters appeared on his hands, feet, and forehead. Throughout his childhood, he suffered from gastrointestinal disturbances and was notably sensitive to sunlight, developing red, swollen skin reactions upon exposure.

**Figure 1:**
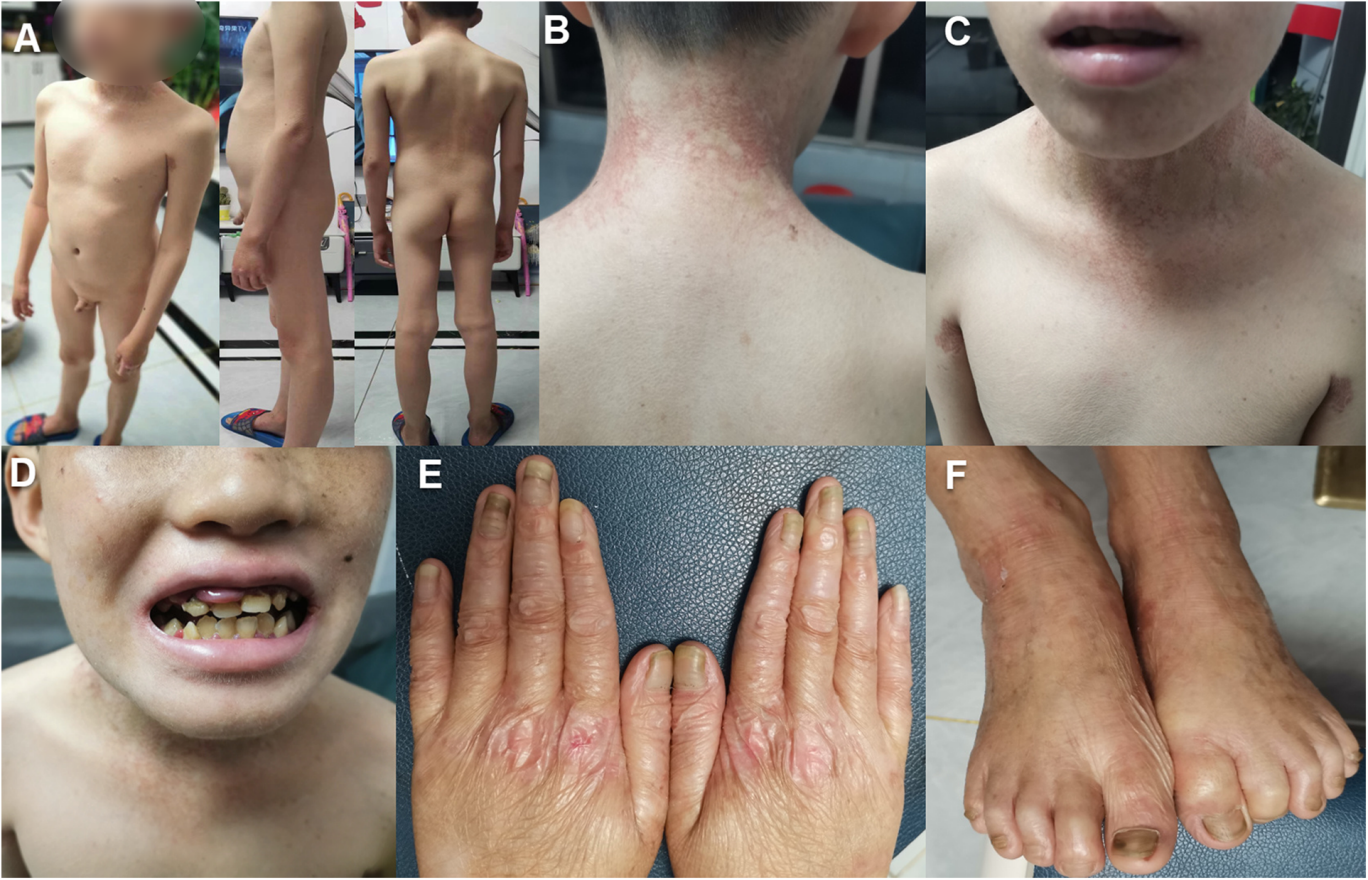
The clinical features of the proband with *FERMT1* variation: **(A–C)**. The patient exhibits generalized xerosis, erythematous patches in the neck, axillary, and lumbar regions, along with hyperpigmentation changes. **(D)** The patient demonstrates malocclusion, irregular spacing between teeth, atypical dental morphology, swollen gums, and angular cheilitis. **(E,F)**. There is skin atrophy on the dorsal surfaces of the hands and feet, presenting with cigarette paper-like wrinkles, dystrophic nails, and a thickened, elongated stratum corneum.

Upon examination, we noted a range of dermatological issues, including generalized dryness, thickened skin, particularly with fissuring on the soles and palms, a waxy appearance of the palms, hyperpigmentation of the skin in the neck and axillary areas, and abnormalities of the fingernails and toenails (Figure 1). However, his sight, neurological profile, urinary reproductive system, and mental development were all normal. Tests including those for heart, liver, kidney function, blood sugar, a thoracoabdominal ultrasound, and a brain MRI showed no abnormalities. The constellation of symptoms aligned with a variety of heritable conditions affecting the integrity of the cutaneous barrier, such as Dyskeratosis Congenita, Rothmund-Thomson Syndrome, Kindler Syndrome, and a spectrum of epidermolysis bullosa resembling the Kindler phenotype. To elucidate the molecular basis, we conducted a genomic investigation using WES.

### Genetic analysis of whole exome sequencing

**Figure 2:**
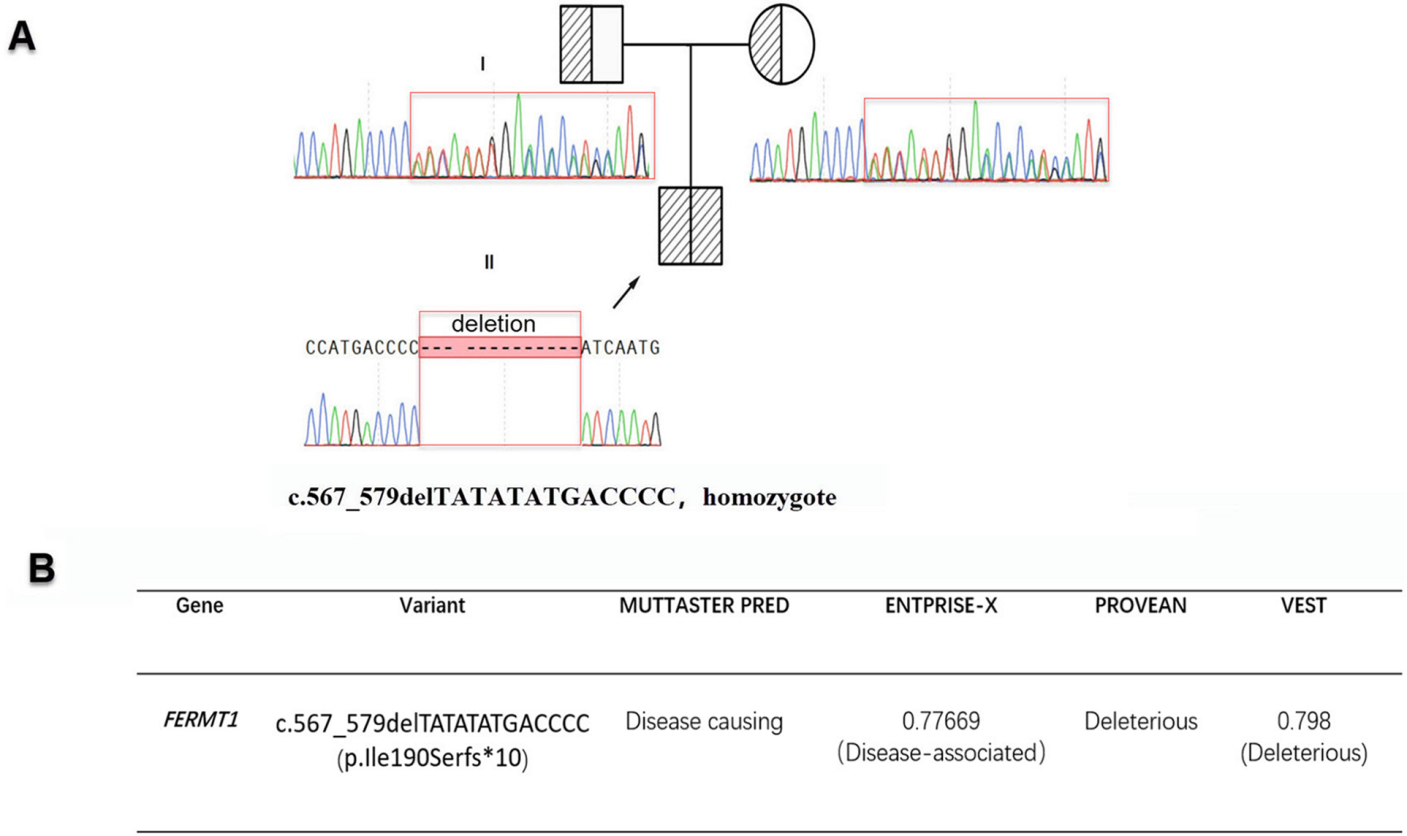
**(A)** The sequencing results for c.567_579delTATATATGACCCC (p.Ile190Serfs*10) along with the patient's family pedigree are displayed. Sanger sequencing confirms c.567_579delTATATATGACCCC (p.Ile190Serfs*10) was inherited from parents, with the proband highlighted by a black arrow. **(B)** The functional implications of c.567_579delTATATATGACCCC (p.Ile190Serfs*10) were assessed using five different computational tools.

Exome capture kit was employed for whole-exome sequencing of the patient's genomic DNA. This enriched for exons within a 67M capture region, achieving an average Q30 score of ≥95%. We attained uniform base distribution with negligible GC bias and generated 20.1G of clean data, representing 98.5% coverage of the targeted exome region (at least 20× coverage). WES identified 56,378 variants, including 14,490 amino acid alterations. Utilizing TGex software (LifeMap Sciences, USA), variants in six genes (*FLG, PLEC, ABCA12*, *COL7A1*, *KRT2 and FERMT1*) linked with the patient's phenotype were detected in OMIM-listed genes. Subsequently, after filtering based on population frequency, inheritance patterns, and pathogenicity, the homozygous deletion c.567_579delTATATATGACCCC (p.Ile190Serfs*10) in the *FERMT1* gene emerged as the most probable causative variant. Sanger sequencing confirmed its parental origin (Figure 2A). This variant was absent from major databases including the 1,000 Genomes Project, the Human Gene Mutation Database, ClinVar, and LOVD, satisfying the PM2 criterion per the ACMG guidelines. The mutation c.567_579delTATATATGACCCC (p.Ile190Serfs*10) is located within the nonsense-mediated mRNA decay (NMD) region. This frameshift mutation directly disrupts the gene's coding sequence, potentially preventing the expression of a functional protein. As a loss-of-function (LOF) variant, it qualifies as a PVS1 (Pathogenic Very Strong) indication. The ACMG/AMP guidelines for interpreting sequence variants were used to assess pathogenicity. According to these guidelines, the frameshift variant observed in the patient is classified as “likely pathogenic” (PVS1 + PM2-supporting), a term synonymous with “pathogenic variant” in a clinical setting. Consequently, the variant is appropriate for clinical diagnosis and decision-making.

### Literature review

To understand the genetic characteristics and clinical features of Kindler Syndrome in the Chinese population, this study included data from OMIM (https://www.omim.org/), Wan Fang (https://www.wanfangdata.com.cn/index.html), CNKI (https://c61.oversea.cnki.net/), and PubMed (https://pubmed.ncbi.nlm.nih.gov/) databases to conduct a literature review on the clinical phenotypes and genetic backgrounds of *FERMT1* variant patients in China (Table 1). According to the statistics, to date, a total of 12 cases of Kindler Syndrome have been reported in the Chinese population.

**Table 1:** Summary of clinical and molecular features of all patients with *FERMT1* variants in Chinese.

Patients	P1 (24)	P2 (25)	P3 (26)	P4 (27)	P5 (28)	P6 (29)	P7 (29)	P8 (30)	P9 (31)	Family 1 (32)			Our study	Statistics
										P10	P11	P12		
Gender	Male	Male	Male	Female	Male	Female	Female	Male	Male	Female	Female	Male	Male	Male: female = 1.60:1.00
Age	38	12	33	7	13	11	30	23	24	15	17	0.67	12	18.13 ± 10.40
Age at onset	childhood	1y	0	neonatal period	2y	N/A	N/A	2y	3d	7d	N/A	8d	0	7.58 ± 10.23 m
Poikiloderma	+	+	+	+	+	+	+	+	+	+	+	N/A	+	100.00%
Dermal atrophy	+	+	+	+	+	+	+	+	+	+	+	+	+	100.00%
Blistering	+	+	+	+	+	+	+	+	+	+	+	+	+	100.00%
Photosensitivity	+	+	+	+	+	+	+	+	+	+	+	N/A	+	100.00%
Oral damage	+	−	+	+	−	N/A	N/A	+	+	−	+	N/A	+	70.00%
Palmoplantar hyperkeratosis	+	+	+	+	+	+	+	+	+	−	+	N/A	+	91.70%
Abnormality of finger/toe	+	−	+	+	+	+	+	+	+	−	−	N/A	+	75.00%
Dysphagia	+	−	+	+	N/A	N/A	N/A	−	N/A	−	−	N/A	−	37.50%
Eye abnormalities	+	−	+	N/A	N/A	N/A	N/A	+	N/A	−	+	N/A	−	57.14%
Constipation	+	−	N/A	+	N/A	N/A	N/A	−	−	N/A	N/A	N/A	+	50.00%
Nail abnormalities	+	−	+	+	−	N/A	N/A	+	N/A	+	+	N/A	+	77.78%
Mutation type	missense/frameshift	frameshift	frameshift	exon deletion	missense	nonsense	frameshift	exon deletion	splice site	N/A	N/A	N/A	frameshift	Lof: 81.82%
Exon or intron	5 (Exon)	8 (Exon)	15 (Exon)	1–7 (Exon)	11 (Exon)	3 (Exon)	3 (Exon)	7–9 (Exon)	9 (Intron)	N/A	N/A	N/A	4 (Exon)	Exon: intron = 12:1
Mutation	c.671C > A c.676 del C	c.994_995 del CA	c.1885_1901 del 17bp	exons 1–7 del 17,252 bp	c.1343T > A	c.193C > T c.277C > T	c.220delC	exons 7–9 del 3,017 bp	IVS-9 + 1 G > A	N/A	N/A	N/A	c.567_579 del TATATATGACCCC	−
Consanguineous	+	N/A	−	+	−	−	+	+	+	−	−	−	−	41.67%
Family history	−	−	−	−	−	−	−	−	−	+	+	+	−	23.08%

N/A, not applicable.

## Discussion

KS is an autosomal recessive genetic disorder first described by Theresa Kindler in 1954, detailing the case of a 14-year-old girl with a history of childhood acneiform bullae who developed acrodermatitis and photosensitive epidermolysis bullosa (3). Since this initial case, KS has been identified in individuals from diverse ethnic backgrounds across the world (18–20). In 2003, genome linkage analysis and whole-genome sequencing facilitated the discovery of the mutation causing KS. Researchers located this mutation in a gene initially known as KIND1 but subsequently renamed *FERMT1* (21). The *FERMT1* gene is responsible for the production of kindlin-1, a protein predominantly present in the skin and colon (22) and essential for cell adhesion and migration. Notably, scientists have identified four distinct *FERMT1* transcripts, each encoding a distinct kindlin-1 isoform involved in cytoskeletal signaling and cell-to-cell adhesion.

**Figure 3:**
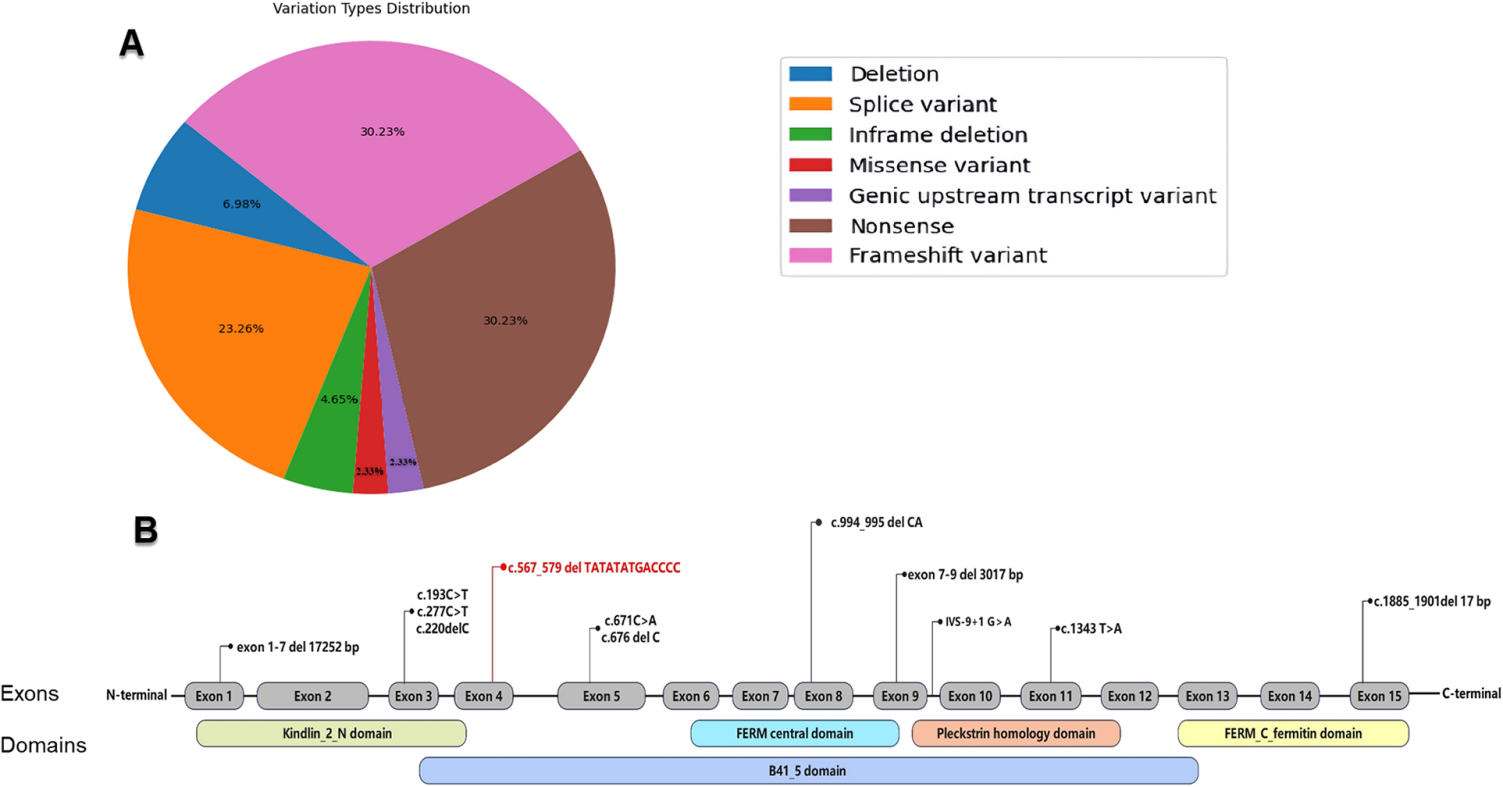
**(A)** Distribution of pathogenic and likely pathogenic variation types in the *FERMT1* as recorded in the ClinVar database. **(B)** Reported *FERMT1* variations in the Chinese population (variation highlighted in red is those reported in the present study).

Presently, the ClinVar database (https://www.ncbi.nlm.nih.gov/clinvar) recognizes 44 pathogenic or likely pathogenic variants connected to KS (Figure 3A). Among these, nonsense and frameshift variants are the most frequently reported, constituting 30.23% each, with splice site variants following at 23.26%. Variants of other types occur less frequently.

Clinically, KS presents with varying degrees of severity, often within the same family, which complicates diagnosis and treatment (refer to Table 1, Family 1). It severely affects the health and quality of life of patients through its skin manifestations and related complications. For individuals with KS, early diagnosis and treatment are critical to prevent further complications and enhance their quality of life.

To our knowledge, this is the inaugural report of KS caused by the c.567_579delTATATATGACCCC (p.Ile190Serfs*10) frameshift variant in the *FERMT1* gene. The clinical symptoms observed in the patient, including skin fragility, blistering, progressing hyperkeratosis, photosensitivity, and mucosal symptoms like gingival hyperplasia and constipation, align with KS's diagnostic criteria as proposed by Angelova-Fischer et al. (Supplementary Table S1) (23). However, it must be emphasized that genetic analysis remains the gold standard for diagnosing Kindler Syndrome. This case emphasizes the significance of clinical assessment coupled with WES in diagnosing rare genetic disorders. Among Chinese patients with KS, there are 13 patients with 12 *FERMT1* variants (refer to Table 1 and Figure 3B) (24–32), predominantly loss-of-function (LoF) types comprising 81.82% of cases. The apparent male dominance (1.61:1 ratio) in our research cohort likely reflects the small sample size rather than a genuine sex-linked difference, given KS's autosomal recessive inheritance. The age of onset for KS ranges broadly, from birth to 38 years, with most cases manifesting in early childhood, occasionally at birth. Patients consistently exhibit KS's defining features such as poikiloderma, skin atrophy, blistering, and photosensitivity, with the latter often diminishing after puberty.

Pathogenesis related to skin fragility and blister formation in KS is associated with Kindlin-1 dysfunction, which causes abnormal integrin activation and disrupts keratinocyte adhesion, the cellular cytoskeleton, and signaling pathways, leading to enhanced vulnerability and fragility of keratinocytes and subsequent blister development (33). The prevalence of blisters usually decreases around ages 10 to 12 years (21, 34). Additional clinical features can include oral lesions (in 70% of cases), palmoplantar keratoderma (91.70%), limb anomalies (75%), dysphagia (37.50%), eye-related symptoms (57.14%), constipation (50%), and nail dystrophy (77.78%). Techanukul et al. have postulated a potential link between gastrointestinal manifestations of KS and variants within exons 2–7 of the *FERMT1* gene, which is supported by the evidence in Table 1 (35).

As an autosomal recessive condition, KS tends to occur more often in populations with high consanguinity rates. Within our study group, 23.08% of KS patients reported a family history of the condition, and 41.67% were offspring of consanguineous unions, which increases the likelihood of carriers for pathogenic *FERMT1* variants among parents. Additionally, there is heightened risk of malignancy in KS patients, with the cumulative risk of squamous cell carcinoma (SCC) escalating with age (14). The morbidity and mortality linked to KS are primarily due to complications such as mucosal stenosis, secondary infections, extensive blistering, and malignancies (19, 36, 37). Consequently, early diagnosis and management are imperative to reduce morbidity and mortality rates in these patients.

According to the ClinVar database, there are 44 identified pathogenic or probably pathogenic variants of the *FERMT1* gene, encompassing missense, nonsense, splice site, frameshift, and deletion types. These variations are predominantly present within exons, while a minority affect intronic sequences, and no specific mutational hotspots have been confirmed. Milder clinical signs and delayed onset of complications have been associated with pathogenic missense and in-frame deletion variants in *FERMT1* (1). The frameshift variant identified in our patient introduces a premature termination codon (PTC). In some cases, such a mutation may result in a truncated protein that retains partial functionality. However, this scenario is not common and requires analysis based on specific circumstances. Generally speaking, mRNA containing a PTC is typically recognized and degraded by nonsense-mediated decay (NMD), preventing the synthesis of the protein altogether. NMDEscPredictor indicated that the variant is likely to undergo NMD, lead to mRNA degradation through. Consequently, a frameshift variant could impair or eliminate the protein's inherent biological activity, which may correlate with the patient's severe and classic Kindler Syndrome (KS) cutaneous phenotype.

Moreover, accumulating evidence suggests a role for *FERMT1* in tumor proliferation, metastasis, apoptosis, and angiogenesis (38, 39). *FERMT1* deficiency may correlate with heightened cancer risk, underlining the necessity for ongoing patient monitoring to facilitate the early identification and treatment of potential malignancies. The management of KS necessitates a collaborative, multidisciplinary strategy, emphasizing meticulous skin care, stringent oral hygiene, and malignancy monitoring, with sun protection being vital due to the patients’ photosensitivity. Regular dental check-ups are pivotal in addressing periodontal concerns, and gastrointestinal assessments are integral to the effective management of potential strictures among other complications.

## Conclusions

In this study, we employed Whole Exome Sequencing (WES), Sanger sequencing, and bioinformatics analysis to discover a novel likely pathogenic variant in the *FERMT1* gene in a Chinese adolescent male with KS, thereby expanding the known spectrum of *FERMT1* variants. Additionally, we reviewed and analyzed the clinical manifestations and molecular genetic characteristics of KS patients reported in the Chinese population, which helps to deepen our understanding of the genetic landscape of KS in different populations. The significance of this discovery lies in its potential to improve the genetic diagnosis and clinical management of KS, providing a more comprehensive global perspective on this rare inherited skin disorder.

Despite the limitations of our study, such as a report of a single case, we recognize the need for further research. Future research directions include increasing the sample size and conducting more functional experiments to determine the effects of the mutation. These expanded studies will not only enhance our understanding of the genetics and phenotypic spectrum of KS but also elucidate the functional consequences of *FERMT1* variants. Ultimately, this will enhance our ability to diagnose, manage, and develop targeted therapies for KS.

## Data Availability

The original contributions presented in the study are included in the article/Supplementary Material, further inquiries can be directed to the corresponding author.
