# Supplementary material for: Identification of a novel FERMT1 variant causing kindler syndrome and a review of the clinical and molecular genetic features in Chinese patients
Source: Front Pediatr. 2024 Sep 6;12:1425030. doi: 10.3389/fped.2024.1425030 (PMC11415864; doi:10.3389/fped.2024.1425030)
Supplement: Supplementary file 1 [file Table1.pdf]

Supplementary Table S1 The diagnostic criteria for Kindler Syndrome (KS) proposed by Angelova-Fischer

Criteria		
**Major criteria**	**Minor criteria**	**Associated findings**
1. Acral blistering in infancy and childhood	1. Syndactyly	1. Nail dystrophy
2. Progressive poikiloderma	2. Mucosal involvement: urethral, anal, esophageal, and laryngeal stenosis	2 Ectropion of the lower lid
3. Skin atrophy		3. Palmoplantar keratoderma
4. Abnormal photosensitivity		4. Pseudoainhum
5. Gingival fragility and/or swelling		5. Leucokeratosis of the lips
		6. Squamous cell carcinoma
		7. Anhidrosis/hypohidrosis
		8. Skeletal abnormalities
		9. Poor dentition/dental caries/periodontitis

Notes:
Major criteria: 4 major criteria present = certain diagnosis; 3 major and 2 minor criteria present = probable diagnosis; 2 major and 2 minor criteria present = likely diagnosis.
Minor criteria: These criteria are considered less specific for KS than the major criteria.
Associated findings: These findings may be present in patients with KS, but they are not specific for the disease.
